# Supplementary material for: Potential Candidate Molecule of Photosystem II Inhibitor Herbicide—Brassicanate A Sulfoxide
Source: Int J Mol Sci. 2024 Feb 18;25(4):2400. doi: 10.3390/ijms25042400 (PMC10889811; doi:10.3390/ijms25042400)
Supplement: Supplementary file 1 [file ijms-25-02400-s001.zip › ijms-2862551-supplementary.pdf]

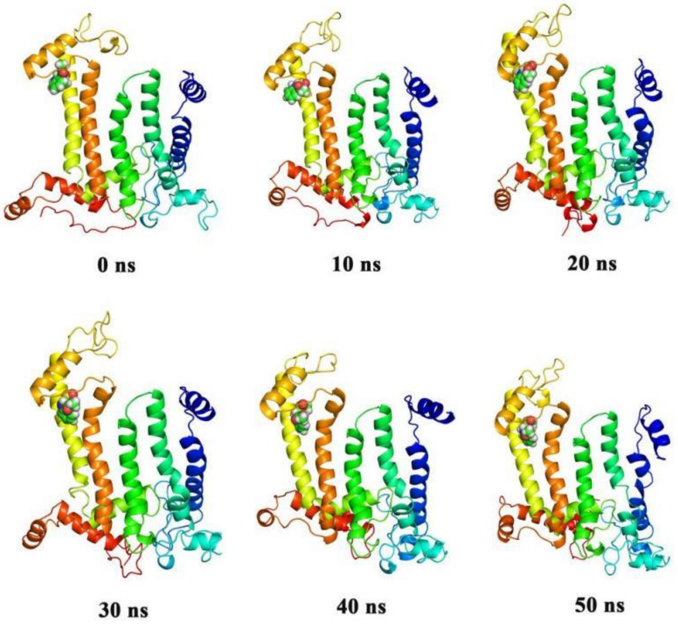

Figure S1. A dynamic simulation snapshot of brassicanate A sulfoxide interacting with PSBD1 over a period of 50 ns.

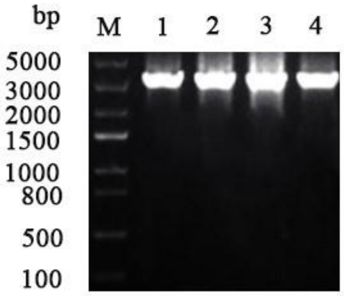

Figure S2. PCR identification of PSBD1 recombinant Bacmid. The numbers represent different individual colonies, M, DL 5000.

Table S1. Plasmid and primer used in this study.

Plasmid	Primer
pFastBacTM1	M13F:TGTAAAACGACGGCCAGT<br>M13R:CAGGAAACAGCTATGACC
